# Anticoagulant Utilization and Cost Analysis among Cardiology Inpatients in a Tertiary Care Teaching Hospital of Western Nepal

**DOI:** 10.1155/2020/8890921

**Published:** 2020-11-23

**Authors:** Sabina Sankhi, Nirmal Raj Marasine, Parbati Thapa, Nim Bahadur Dangi

**Affiliations:** Pharmaceutical Sciences Program, School of Health and Allied Sciences, Pokhara University, Pokhara 30, Kaski, Nepal

## Abstract

**Introduction:**

Anticoagulants have a wide spectrum of use and risks associated with their therapy due to their narrow therapeutic range. This study aimed to evaluate the anticoagulant utilization and cost analysis in patients admitted to the cardiology ward of a tertiary care hospital in western Nepal.

**Methods:**

A prospective cohort study was conducted in patients admitted to the cardiology ward of Manipal Teaching Hospital (MTH), Pokhara, Kaski, Nepal, from August to November 2019. All patients (*n* = 132) aged ≥18 years of either gender receiving anticoagulants for any indication in the cardiology ward were included in the study. Anticoagulant utilization, the average prescribed daily dose (PDD/DDD) and the cost of anticoagulant per patient were calculated. Descriptive statistics were performed using IBM-SPSS 20.0.

**Results:**

Acute coronary syndrome (66.67%) was a common indication, unfractionated heparin + enoxaparin (45.45%) and enoxaparin (27.3%) were the most frequently prescribed anticoagulants. The performance of monitoring parameters such as international normalized ratio (INR), prothrombin time (PT), activated partial thromboplastin time (aPTT), and renal function test were consistent with the American College of Chest Physician (ACCP) guidelines. The average prescribed daily dose of anticoagulants was 1.3 (unfractionated heparin), 2.25 (enoxaparin), 0.5 (warfarin), and 1.0 (dabigatran). Heparin was associated with the majority of cases of drug interactions (52 cases). Enoxaparin was the most expensive of all the anticoagulant drug classes. The median (IQR) cost of anticoagulants used per patient was US$79.92 ($46.32).

**Conclusion:**

Our study suggests that the utilization of unfractionated heparin and enoxaparin and the cost of anticoagulants per patient were higher in the patients admitted to the cardiology ward of the hospital.

## 1. Introduction

Drug utilization has been defined as “the marketing, distribution, prescription, and use of drugs in society, with special emphasis on the resulting medical, social, and economic consequences.” It helps healthcare systems to understand, interpret, and improve the prescription, administration, and use of medications, which in turn improves patient therapeutic outcomes [1].

About 7 million people worldwide are taking anticoagulants for the management of their chronic diseases [2]. Anticoagulants are frequently prescribed drugs in deep vein thrombosis, pulmonary embolism, myocardial infarction, unstable angina, atrial fibrillation, acute coronary syndrome, rheumatic heart disease, vascular surgery, and prosthetic heart valve, in both inpatient and outpatient settings [3]. They are divided into oral (coumarin derivatives: warfarin and acenocoumarol) and parenteral agents [indirect thrombin inhibitors: unfractionated heparin (UFH) and low molecular weight heparin (LMWH), such as enoxaparin, dalteparin, and direct thrombin inhibitor, lepirudin]. The latter is widely used for short-term therapy, basically when rapid anticoagulation is required [4].

Historically, anticoagulation therapy with warfarin has been the cornerstone of oral anticoagulant therapy worldwide [5]. The ability to monitor the degree of anticoagulation, reversibility of effects, and low-cost generic availability of warfarin appealed both patients and physicians for its use. Despite its effectiveness in reducing thromboembolic events, it has several drawbacks, including a narrow therapeutic effect, a delayed onset and offset of action, complex dosing with genetic variances, potential drug interactions, and routine monitoring of international normalized ratio (INR), prothrombin time (PT), activated partial thromboplastin time (aPTT), platelet count along with patient-specific dose adjustments [6–8]. Additionally, these drug classes (both warfarin and unfractionated heparin) have a high chance of causing adverse effects such as bleeding, heparin-induced thrombocytopenia, osteoporosis, hemorrhagic stroke, and even death [9]. Lack of proper monitoring can result in inappropriate dosing and complications such as bleeding and thrombosis. This, as a whole, leads to increased duration of hospital stay, increased overall healthcare costs, decreased therapeutic outcome, and eventually increased mortality [10]. All these incidents motivated the development of newer oral anticoagulant therapy, called non-vitamin K antagonist oral anticoagulant (NOAC).

Non-vitamin K antagonist oral anticoagulants (NOACs), such as direct factor Xa inhibitors: rivaroxaban and apixaban, and direct thrombin inhibitor: dabigatran, are newer agents approved by current guidelines for the prevention of stroke and systemic embolism in patients with atrial fibrillation and patients with venous thromboembolism [6–8]. In contrast to warfarin, these newer agents do not require routine monitoring of anticoagulant effects because of their more predictable pharmacological profiles, rapid onset and offset of action, and fewer drug-drug and drug-food interactions [9, 11].

There is a paucity of data on anticoagulant utilization and associated costs to the patients, especially in inpatients in Nepal. Evidence suggests that physicians familiarity, clinical experience, and efficacy have been influencing the prescription of anticoagulants. Determining the utilization of anticoagulants within a broader patient population could have a positive effect on patient outcomes and overall healthcare costs. Therefore, we aimed to assess the anticoagulant utilization and cost in patients admitted to the cardiology ward of a tertiary care teaching hospital in western Nepal.

## 2. Methodology

### 2.1. Ethics

Ethical approval for this study was obtained from the Institutional Review Committee (IRC) of Pokhara University Research Center (PURC) (ref. no. 20/076/077). Prior permission to conduct the study was obtained from the Manipal Teaching Hospital, Pokhara, Nepal. The patients or caretakers were fully informed about the nature and purpose of the study in Nepali language, and their written consent was obtained prior to data collection. Personal details of the patients were kept confidential, and anonymity was maintained.

### 2.2. Study Design and Population

A prospective cohort study was conducted between August 2019 and October 2019 among 132 patients in the cardiology ward of Manipal Teaching Hospital (MTH), Pokhara, Nepal. All patients aged ≥18 years of either gender, receiving anticoagulants for any indication in the cardiology ward and those willing to participate were included in the study. Outpatients, patients admitted in a department other than the cardiology and those diagnosed with mental retardation were excluded from the study.

### 2.3. Data Collection

Data were collected prospectively from the patients' Kardex. All patients were followed up until they stayed in the cardiology ward. Information on demographics (age, gender, occupation, and education), laboratory test results of parameters such as INR for warfarin, PT, and aPTT for UFH and renal function test before using enoxaparin, anticoagulant use (indication, prescribed anticoagulants, generic name, dose, dosage form, frequency, and route of administration of antibiotic), length of hospital stay, and anticoagulant cost at the time of the study were collected in a well-designed proforma. According to the World Health Organization (WHO), the prescribed daily dose (PDD) is the average dose prescribed according to a representative sample of prescriptions. It gives the average daily amount of a drug that is prescribed and expressed as the PDD:DDD ratio [12].

PDD was calculated as

(1) $$\begin{array}{c} \text{PDD}=\frac{\text{total dose of a drug over a specified period}}{\text{number of days}}. \end{array}$$

The calculated prescribed daily dose was compared with the respective WHO's precalculated defined daily dose (DDD) [13]. The individual anticoagulant cost was calculated by the multiplication of the cost per unit and the number of doses prescribed. The unit price of each anticoagulant used was obtained from the hospital pharmacy.

### 2.4. Data Analysis

The data were entered in Microsoft Excel version 13 and analyzed using IBM-SPSS 20.0 (IBM Corporation, Armonk, NY, USA). Data were expressed as median and interquartile range (IQR), and descriptive statistics were used. The Kolmogorov–Smirnov test was used to determine the normality of the numeric variables. Stockley's drug interactions, 9th Edition [14], was used for analyzing potential drug-drug interactions.

## 3. Results and Discussion

Among the total of 132 patients, more than half (77, 58.3%) were male. One-fourth of the study participants (34, 25.8%) aged 61–70 years. The most frequently reported occupation was housewife or unemployed (70, 53%). Fifty-two (39.4%) of them were illiterate, and 38 (28.8%) of them had an elementary level of education, as shown in Table 1.

This represented that this patient population was at a higher risk of developing cardiovascular diseases. This finding was consistent with that of another study conducted in Pakistan [15]. Increased loneliness and stress in unemployed or housewife, business/job holders, poor control of pre-existing cardiovascular risk factors in farmers, and prolonged stable postures (sitting or standing) in builders, drivers, cooks, and tailors may increase the risk of cardiovascular diseases. The length of hospital stay ranged from 1 to 15 days, with a median (IQR) of 6 (3) days.

The majority of the patients (81, 61.35%) were prescribed more than one anticoagulant medication, where unfractionated heparin and enoxaparin comprised a major proportion (60, 45.45%), followed by enoxaparin and warfarin (21, 15.90%). Enoxaparin (36, 27.3%) was the most frequently prescribed monotherapy compared to all other anticoagulants. In both monotherapy and combination therapy, use of enoxaparin was common. This might be due to its greater bioavailability, longer plasma half-life, predictable anticoagulant effect, and lower incidence of osteoporosis and heparin-induced thrombocytopenia [16]. The current availability of NOAC in Nepal along with the lack of clinical experience among physicians, higher cost, unavailability of antidotes, and contraindications in patients with severe kidney or liver disease might be the reason for the lower use of dabigatran (3.78%) than warfarin, which was used in monotherapy as well as in combination therapy in our study [17]. This was comparably lower than that of the studies from Canada [18] and Turkey [19], where dabigatran was used in 46.8% and 24% of the patients, respectively, and warfarin was the mainstay of the therapy (53.2% and 73%, respectively).

As in a study done in Iran [10], acute coronary syndrome was the common clinical condition followed by congestive heart failure for which an anticoagulant was prescribed.

Our study showed that there was a high variation in the cost of these utilized anticoagulants. The total cost of anticoagulants prescribed in all the patients was $8155.09, and the average individual anticoagulant cost was US$79.92, but it varied from US$0.03 to US$126.24, as illustrated in Table 2. For example, enoxaparin 60 mg/ml was prescribed twice a day for 5 days. Enoxaparin 60 mg/ml costs NRs 911.52 during the study period. Within 5 days of hospital stay, 10 doses were used in patients, which cost 911.52 × 10 = Rs. 9115.20 ($79.92) for that individual. Similarly, during 1 day of hospital stay, only warfarin 2 mg was prescribed once daily. Warfarin 2 mg costs NRs 3 per tablet, and as only one tablet was used by the patient, the anticoagulant cost of that patient became 3 × 1 = 3 ($0.03). In similar way, the cost was calculated in patients prescribed with more than one anticoagulant. Enoxaparin was the most expensive anticoagulant of all and further increases the burden when added to the cost of diagnosis and monitoring. A study in the United Kingdom [20] reported that the patients spent approximately US$726.58 on total anticoagulant cost per year. On the contrary, global comparisons of anticoagulant utilization costs could be often misleading due to the alteration of drug prices globally.

As per the ACCP guidelines, monitoring parameters such as PT/INR/aPTT and renal function tests are important factors for monitoring and controlling anticoagulant use. During the study period, baseline PT/INR/aPTT was performed in the majority of the patients (87.9%). In the case of enoxaparin, a renal function test is a must since they are renally cleared drugs and was performed in 94.87% of enoxaparin users [6–8], as depicted in Table 3.

In contrast, laboratory monitoring of PT/INR/aPTT was not performed in enoxaparin users, as they do not prolong aPTT/clotting time. Likewise, aPTT was monitored for unfractionated heparin every 6 hours and on every second day of warfarin therapy until the therapeutic goal (2-3) was reached. Moreover, it is recommended to carry out a kidney function test before initiation of any NOAC, to determine if dose reductions are necessary [21]. However, regular monitoring of PT/INR for dabigatran as like warfarin (i.e., on a 24-hour basis) was observed in our study, which might contribute to an increase in cost burden to the patients. In addition, in many patients, magnetic resonance imaging (MRI), angiography, and computerized tomography (CT) scans were performed to detect the presence of clots or thrombus formation.

From the calculated PDD of each anticoagulant when compared with WHO's provided optimal dose, unfractionated heparin was prescribed more than the optimal dose, enoxaparin was prescribed more than double the optimal dose, and warfarin was prescribed half the optimal dose. In contrast, only dabigatran was prescribed within the WHO's provided optimal dose, as illustrated in Table 4.

These findings of our study were consistent with those of a study from our neighboring country, India [3], where unfractionated heparin and enoxaparin were prescribed above the optimal dose (1.3 and 1.1, respectively), warfarin was prescribed below the optimal dose (0.53), and NOAC (rivaroxaban) was prescribed within the WHOs provided optimal dose (1.0). The utilization of enoxaparin was more than twice in our study. This fluctuation in the utilization of anticoagulants might be due to variations in diagnosis, age, weight, and severity of the disease, which resulted in a marked difference in the dosing quantity and PDD differed from WHO's DDD [12].

Of the total 65 cases of potential drug interactions analyzed during the study period, unfractionated heparin was the drug commonly involved in 52 (80%) of the cases (unfractionated heparin + aspirin: 44 and unfractionated heparin + telmisartan: 8), as depicted in Table 5. Likewise, in a similar study conducted in Ethiopia [22], unfractionated heparin was found involved in 35% of the potential drug interaction cases and enoxaparin in only 10.7% of the cases.

There are a number of limitations to this study. We explored the anticoagulant utilization pattern over a period of three months. Hence, the influence of seasonal variations on disease pattern and anticoagulant utilization could not be considered. Similarly, this study failed to examine confounding factors such as diet patterns, which could have influenced the significance of various factors on the utilization pattern. Likewise, the total healthcare costs of the individual patient was outside the scope of this study, and therefore we were only able to calculate the cost for anticoagulant use. Despite these limitations, this study provides an insight into the anticoagulant use among inpatients in the cardiology ward and the costs associated with it. The findings might be beneficial for policy formulation of anticoagulants use in Nepal. This is probably the first study on anticoagulants in the inpatient setting and first to calculate the prescribed daily dose for anticoagulants in Nepal. Thus, it can serve as baseline data for conducting studies in a similar set up in near future to identify the trends in drug consumption over the years.

## 4. Conclusion

Our study suggests that the utilization of anticoagulants is not to the optimum except for dabigatran. Especially, enoxaparin use was more than twice as directed by the WHO. Moreover, anticoagulant costs per patient was also high. This highlights the need for development and implementation of standard treatment guidelines, protocols, and subsequent pharmacoeconomic evaluation of anticoagulation therapy for better patient outcomes and making treatments cost-effective and affordable to every socioeconomic community.

## Figures and Tables

**Table 1:** Sociodemographic characteristics of patients (*n* = 132).

Characteristics	Categories	*n* (%)
Age	18–40	9 (6.8)
	41–50	11 (8.3)
	51–60	31 (23.5)
	61–70	34 (25.8)
	71–80	33 (25.0)
	>80	14 (10.6)

Gender	Male	77 (58.3)
	Female	55 (41.7)

Occupation	Business	21 (15.9)
	Service	12 (9.1)
	Agriculture	14 (10.6)
	Housewife or unemployed	70 (53.0)
	Others	15 (11.4)

Education	Illiterate	52 (39.4)
	Literate but not attended formal classes	19 (14.4)
	Elementary level	38 (28.8)
	Secondary level	17 (12.9)
	Undergraduate and above	6 (4.5)
^†^Length of hospital stay (days)	1 to 15	6 (3)

^†^Median (IQR) instead of *n* (%); IQR: interquartile range.

**Table 2:** Anticoagulant use and cost in the cardiology unit (*n* = 132).

Characteristics	Categories	*n* (%)
Prescribed anticoagulants	Unfractionated heparin (UFH)	4 (3.03)
	Unfractionated heparin + enoxaparin	60 (45.45)
	Enoxaparin	36 (27.3)
	Enoxaparin + warfarin	21 (15.90)
	Warfarin	6 (4.54)
	Dabigatran	5 (3.78)
Indication of anticoagulants use	Acute coronary syndrome (ACS)	88 (66.67)
	Congestive heart failure (CHF)	40 (30.30)
	Rheumatic heart disease (RHD)	4 (3.03)
^†^Individual drug cost	$0.03 to $126.24	$79.92 ($46.32)

^†^Median (IQR) instead of *n* (%); IQR: interquartile range. 1$ (USD) = 114.05 Nepalese rupees (NRs) during the study period.

**Table 3:** PT/INR/aPTT test and renal function test performance before enoxaparin

Characteristics	Categories	*n* (%)
Whether PT/INR/aPTT was performed or not? (*n* = 132)	No	16 (12.1)
	Yes	116 (87.9)

RFT performance before enoxaparin (*n* = 117)	No	6 (5.13)
	Yes	111 (94.87)

PT: prothrombin time; INR: international normalized ratio; aPTT: activated partial thromboplastin time; RFT: renal function test.

**Table 4:** Average prescribed daily dose (PDD/DDD) of anticoagulants.

Drug	ATC code	DDD	PDD	PDD/DDD
Unfractionated heparin	B01AB01	10 000 U (P)	13 333 U	1.3
Enoxaparin	B01AB05	2000 U (P)	4500 U	2.25
Warfarin	B01AA03	7.5 mg (O)	3.75 mg	0.5
Dabigatran	B01AEO7	0.22 g (O)	0.22 g	1

ATC: Anatomical Therapeutic Chemical; DDD: defined daily dose; PDD: prescribed daily dose; P: parenteral; O: oral.

**Table 5:** Potential drug-drug interactions (*n* = 132).

Drug interaction found	Number of cases	Effect
Unfractionated heparin + aspirin (moderate)	44	May potentiate the risk of bleeding
Unfractionated heparin + telmisartan (moderate)	8	May potentiate the risk of hyperkalemia
Enoxaparin + aspirin (major)	6	May potentiate the risk of bleeding complications
Enoxaparin + clopidogrel (major)	5	May potentiate the risk of bleeding
Enoxaparin + telmisartan (moderate)	2	May increase the risk of hyperkalemia

## Data Availability

The raw data used to support the findings of this study are made available from the corresponding author upon request.
